# Contribution of Dietary Supplements to Nutritional Adequacy in Race/Ethnic Population Subgroups in the United States

**DOI:** 10.3390/nu9121295

**Published:** 2017-11-28

**Authors:** Jeffrey B. Blumberg, Balz Frei, Victor L. Fulgoni, Connie M. Weaver, Steven H. Zeisel

**Affiliations:** 1 Antioxidants Research Laboratory, Jean Mayer USDA Human Nutrition Research Center on Aging, and the Friedman School of Nutrition Science and Policy, Tufts University, Boston, MA 02111, USA; jeffrey.blumberg@tufts.edu; 2 Linus Pauling Institute and Department of Biochemistry & Biophysics, Oregon State University, Corvallis, OR 97331, USA; balz.frei@oregonstate.edu; 3 Nutrition Impact, LLC, Battle Creek, MI 49014, USA; 4 Department of Nutrition Science, Purdue University, West Lafayette, IN 47907, USA; weavercm@purdue.edu; 5 Nutrition Research Institute, Department of Nutrition, University of North Carolina, Kannapolis, NC 28081, USA; steven_zeisel@unc.edu

**Keywords:** vitamin/mineral supplement, NHANES, micronutrients, non-Hispanic white, non-Hispanic Black, Hispanic, non-Hispanic Asian

## Abstract

The U.S. Centers for Disease Control and Prevention has reported that nutritional deficiencies in the U.S. population vary by age, gender, and race/ethnicity, and could be as high as nearly one third of certain population groups. Based on nationally representative data in 10,698 adults from National Health and Nutrition Examination Surveys (NHANES) primarily from 2009–2012, assessments were made of race/ethnic differences in the impact of dietary supplements on nutrient intake and prevalence of inadequacies. Compared to food alone, use of any dietary supplement plus food was associated with significantly higher intakes of 14 to 16 of 19 nutrients examined in all race/ethnic groups; and significantly (*p* < 0.01) reduced rates of inadequacy for 8/17 nutrients examined in non-Hispanic whites, but only 3–4/17 nutrients (calcium, and vitamins A, D, and E) for other race/ethnic groups. Across race/ethnic groups an increased prevalence of intakes above the Tolerable Upper Intake Level (UL) was seen for 1–9/13 nutrients, but all were less than 5% of the population. In conclusion, use of dietary supplements is associated with increased micronutrient intake, decreased nutrient inadequacies, and slight increases in prevalence above the UL in all race/ethnicities examined, with greater benefits among non-Hispanic whites.

## 1. Introduction

Although adequate intake of essential nutrients is critical for optimal health, many Americans fail to achieve recommended nutrient intake levels. The Dietary Guidelines for Americans 2015–2020 (DGA) [1] recommends consuming nutrient-dense foods as part of a healthy eating pattern and, in some cases, fortified foods and dietary supplements to help meet recommended intakes. The Institute of Medicine’s Food and Nutrition Board has established Estimated Average Requirements (EAR) and Adequate Intakes (AIs) as age- and gender-specific nutrient intake goals [2,3]. The DGA identified potassium, dietary fiber, choline, magnesium, calcium, iron (for certain age/gender groups), and vitamins A, D, E, and C as “underconsumed nutrients”; and vitamin D, calcium, potassium and fiber as "nutrients of public health concern” because low intakes are associated with an increased risk of chronic disease [1].

Intake of dietary supplements has been shown to increase overall nutrient intake and decrease the prevalence of nutrient inadequacy [4]. Taking supplements is a health and lifestyle choice, and the key motivators for consumers appear to be maintenance or improvement in overall health as well as specific health benefits rather than filling nutritional gaps [5,6]. Dietary supplement consumption has increased over time in the United States [7,8] and it has been reported that about 50% adults take dietary supplements and more than 2/3rd of these use multi-vitamin multi-mineral supplements [9,10,11].

A number of studies have investigated race/ethnic differences in adherence with the dietary recommendations and reported a great variance across race/ethnic groups [12,13,14,15,16]. Significant differences in intakes of several micronutrient intakes have also been reported in various population subgroups [17,18,19,20,21,22]. The Second National Report on Biochemical Indicators of Diet and Nutrition in the U.S. Population also found that in 2003–2006 nutritional deficiencies varied by age, gender, or race/ethnicity and could be as high as nearly one third of certain population groups [23]. It is also hypothesized that differential consumption of key nutrients may contribute, at least in part, to the observed disparities in diet-related diseases among these race/ethnic groups. However, only a few studies have investigated race/ethnic differences in the use of dietary supplements [24,25,26,27] and in their effects on prevalence of nutrient inadequacy [28].

The primary objective of this cross-sectional study was to investigate race/ethnic differences in effect of dietary supplements on nutrient intake and prevalence of inadequacies using a large nationally representative data set. This study was part of a broader effort to determine the effect of dietary supplements among diverse US populations [29].

## 2. Materials and Methods

### 2.1. Study Population

The National Health and Nutrition Examination Survey (NHANES), a nationally representative, cross-sectional survey of non-institutionalized, civilian U.S. residents, was used. The data for adults age 19 years and older from NHANES 2009–2010 and 2011–2012 surveys were combined for all analysis except where noted. Race/ethnicity information was self-identified by respondents from a list of 16 groups and the combined sample included 4649 non-Hispanic white (NH-white), 2358 non-Hispanic Black (NH-black), 2625 Hispanic (Mexican Americans and other Hispanics), and 615 non-Hispanic Asian (NH-Asian, data available only for NHANES 2011–2012) participants. Data from pregnant and/or lactating females and from those with incomplete or unreliable records, as judged by the USDA Food Surveys Research Group staff, were excluded. All participants or proxies provided written informed consent and the Research Ethics Review Board at the NCHS approved the survey protocol [30].

### 2.2. Micronutrient Intake from Foods

Two reliable 24-h recall dietary interviews using United States Department of Agriculture’s (USDA) automated multiple-pass method (AMPM) were used to estimate dietary intake [30]. The nutrients intakes from foods were determined using the Food and Nutrient Database for Dietary Studies (FNDDS) 2009–2010 and 2011–2012 [31,32] in conjunction with USDA National Nutrient Database for Standard Reference (SR) releases 24 and 26 [33] for NHANES 2009–2010 and 2011–2012 participants, respectively. 

### 2.3. Micronutrient Intake from Supplements

A dietary supplement questionnaire assessing the usage of vitamins, minerals, botanicals, and other dietary supplements over the past 30 days was administered as part of NHANES household interview and the consumption frequency, the duration, and the amount was recorded for each supplement [34]. The complete product information including labeled dosage or serving size, ingredients, and the amounts of ingredients per serving, was also recorded. The average daily intake of nutrients from dietary supplements was calculated using the supplement consumption frequency and dosage.

### 2.4. Statistics

NHANES survey weights, strata, and primary sampling units were used in all calculations, thus providing nationally representative estimates. The distribution of usual intake including percentiles and percentages meeting cutoffs is estimated using version 2.1 of the National Cancer Institute (NCI) usual intake SAS macro programs [35]. The estimates are generated with two days of dietary data and use day 1 dietary weights in all stages of the estimation process. Balanced repeated replication (BRR) with a Fay adjustment factor of 0.3 is used for variance estimates. The number of BRR replicates equals the smallest multiple of 4 that is greater than the number of NHANES strata in the input dataset for the usual intake. A non-response adjustment based on age, gender and race/ethnicity is made to BRR weights for each replication. The method uses covariates Dietary Reference Intake age group, day of recall, and weekday/weekend flag in the estimation. Supplement usage flag (yes/no) is also included as a covariate when the intake includes both dietary and supplement intake. When intake includes both dietary and supplement intake (30 day supplement intake), the supplement intake is considered to be in long term (usual) form and is added directly to estimated usual intake for the dietary portion to obtain estimated usual intake for diet plus supplements. The NCI method produces percent usual intake less than a preset cutoff during the estimation process. This is done by estimating percent less than cutoff from a Monte Carlo dataset created during the estimation process. The cut-point method of estimating percent less than cutoff is used in all cases except for iron. For iron, published risk curves and numerical integration are used to produce estimates using the probability method. Percentage of the population below the EAR using the cut-point method (except for iron where the probability method was used) for 17 nutrients (calcium, copper, iron, magnesium, phosphorus, selenium, zinc, vitamin A, thiamin, riboflavin, niacin, folate, vitamin B6, vitamin B12, vitamin C, vitamin D, and vitamin E) were determined. The percentage of the population above the AI for 2 nutrients (vitamin K and choline; given an EAR has not been established the percentage of the population with inadequate intakes cannot be determined [36]) were assessed. The percentage of the population above the Upper Tolerable Level (UL) for 13 nutrients (calcium, copper, iron, phosphorus, selenium, zinc, vitamin A as retinol, folate as folic acid, vitamin B6, vitamin C, vitamin D, vitamin E as added alpha tocopherol, and choline) were also assessed. Potassium and sodium were excluded from the present analysis as negligible amounts are found in dietary supplements. All statistical analyses were performed with SAS software (version 9.2; SAS Institute Inc., Cary, NC, USA) and SUDAAN (version 11; Research Triangle Institute; Raleigh, NC, USA). A Z-statistic was used to test significant differences across race/ethnicities and *p* < 0.01 was deemed significant. Data are presented as mean ± SE.

### 2.5. Trial Registration

Not applicable; as this is secondary analysis of publicly released observational data (NHANES 2009–2012).

## 3. Results

### 3.1. Dietary Supplement Usage

Dietary supplement use (mean ± standard error) was reported by 61.1 ± 0.8% of NH-whites, 40.7 ± 1.2% of NH-blacks, 36.6 ± 1.3% of Hispanics and 53.6 ± 3.2% of NH-Asians.

### 3.2. Effect of Supplement Use on Usual Intake of Nutrients

Usual intake of nutrients from food and supplement combined was significantly higher (*p* < 0.01) for all nutrients (except for phosphorus, vitamin K and choline) than from food only (Table 1). However, the difference in magnesium was significant only for NH-whites and the difference in selenium was not significant for Hispanics. Usual intakes of DGA-identified “underconsumed nutrients” increased significantly in the race/ethnic subgroups (range of differences in means across race/ethnic groups) by 8–20% for calcium, 11–21% for iron, 34–55% for vitamin A, 42–114% for vitamin C, 82–218% for vitamin D and 129–236% for vitamin E. The magnitude of increase was generally highest for NH-white and lowest for Hispanic population subgroups.

### 3.3. Effect of Supplement Use on Prevalence of Inadequacy

Consumption of dietary supplements significantly decreased (*p* < 0.01) the prevalence of inadequacy (intakes below EAR) for DGA-identified “underconsumed nutrients” (range of differences in means across race/ethnic groups) by 17–38% for vitamin D and 17–34% for vitamin E for all race/ethnicities; 17–33% for calcium across all race/ethnicities except Hispanics; 14–28% for vitamin A across all race/ethnicities except Asians; and 9% for magnesium and 30% for vitamin C only in the NH-white population subgroup (Table 2). There was also a significant decrease (*p* < 0.01) in % NH-white population with intakes below EAR for vitamin B6 and folate due to dietary supplement intake. There were no significant differences in % population with intakes above AI of vitamin K or choline with supplement intake (plus food) compared to food alone among adults of any race/ethnicity.

### 3.4. Comparison of Prevalence of Inadequacy by Race/Ethnicity

**Figure 1:**
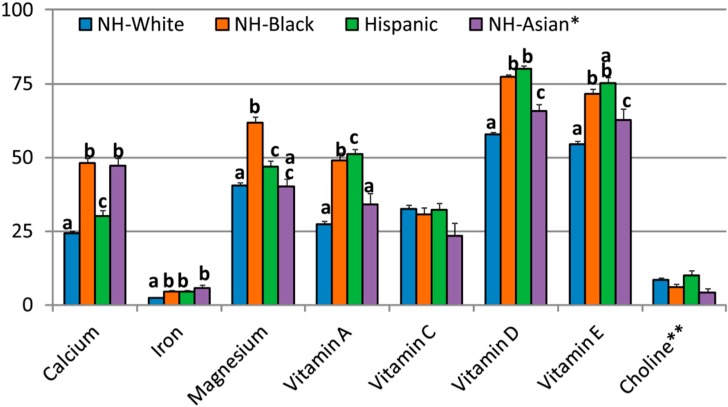
Comparison of percent adult population below Estimated Average Requirement (EAR) or above Adequate Intake (AI) of DGA-identified “underconsumed nutrients” from foods + dietary supplements among different race/ethnicity groups. NHANES 2009–2012 gender combined data. Values are means ± standard error; race/ethnicity groups with different letters within a nutrient are significantly different at *p* < 0.01. * NHANES 2011–2012 only; ** AI Nutrient.

There were significant differences in proportion of population with intakes (from food + supplement) below EAR by race/ethnicity (Table 3, Figure 1). The NH-white population subgroup had a lower prevalence of inadequacy for most nutrients compared to other race/ethnic population subgroups. Among DGA-identified “underconsumed nutrients” (range of differences in means across race/ethnic groups), NH-whites had 20–50% lower prevalence for calcium inadequacy, 48–59% lower prevalence for iron inadequacy, 14–35% lower prevalence for magnesium inadequacy, 44–47% lower prevalence for vitamin A inadequacy, 12–28% lower prevalence for vitamin D inadequacy and 24–27% lower prevalence for vitamin E inadequacy. NH-whites, compared to NH-blacks and Hispanics, also had a higher proportion of population with intakes of vitamin K (from food + supplement) above the AI; NH-Asians had the highest proportion above the AI for vitamin K of all the race/ethnic groups.

### 3.5. Effect of Supplement Use on Prevalence of Intakes above the UL

For food plus supplement as compared to food alone there was a higher (*p* < 0.01) prevalence of intakes above UL for calcium (NH-white and Hispanics), iron (all race/ethnicities except NH-Asians), selenium (NH-white), zinc (all race/ethnicities except NH-Asians), and vitamin A (all race/ethnicities except NH-Asians), folate (all race/ethnicities except NH-Asians), vitamin B6 (all race/ethnicities except NH-Asians), vitamin C (NH-white), and vitamin D (all race/ethnicities except NH-black) (Table 4). However, the actual percentages above the UL were mostly below 2% and were never more than 5% for any nutrient for any race/ethnicity.

## 4. Discussion

The present analysis of recent NHANES (2009–2012) data identified a range of micronutrient inadequacies among US race/ethnic population subgroups, and is the first such analysis to include Non-Hispanic Asians. Dietary supplement use consistently contributed to increased intakes of nutrients and decreased prevalence of inadequacy by population race/ethnic subgroups. 

Use of dietary supplements significantly increased nutrient intakes and decreased the percent population with inadequate intakes (not meeting the EAR) for most nutrients in all race/ethnic population subgroups. Calcium, potassium, iron (adolescent and adult females), magnesium, dietary fiber, choline, and vitamins A, D, E, and C are “underconsumed nutrients” as their intakes for many individuals are below the recommendations [1]. Nutrient deficiencies are associated with increased risks of several adverse health effects including cardiovascular disease, stroke, impaired cognitive function, cancer, eye diseases, poor bone health and other conditions [23,37,38]. Low intakes of calcium, potassium, iron (adult females) dietary fiber, and vitamin D are associated with health concerns [1].

There were also differences in supplement use by race/ethnicities, with a higher incidence of supplement use among NH-white than the other race/ethnicities. NH-white and “Caucasians” have been consistently shown to use more supplements than other race/ethnicities [20,25,26,27,28]. The higher prevalence of supplement use among NH-whites implies higher nutrient intake and lower prevalence of nutrient inadequacy. Race/ethnicity may also influence cultural preferences for certain foods, food combinations, and methods of preparation and therefore may result in race/ethnic differences in dietary intakes from food alone. The present analysis also identified several race/ethnic differences in prevalence of inadequacies for nutrients. It is interesting to note that while there was generally an increased intake associated with dietary supplements across all race/ethnicities, the decrease in prevalence of inadequacy among NH-blacks, Hispanics and Asians were not as large as in NH-white sub-group. It appears the former population subgroups typically start with lower intakes and a lower percentage of those populations are taking dietary supplements. Indeed, the NH-white population subgroup had a significantly lower prevalence of inadequacies for many DGA-identified “underconsumed nutrients” (nutrients where a significant percentage of the U.S. population or specific sub-groups of the population have inadequate intakes) and “nutrients of public health concern” (nutrients for which low intakes are associated with health concerns) than the other race/ethnic population subgroups. Significantly lower intakes of several key nutrients including “underconsumed nutrients” and “nutrients of public health concern” by NH-black and Hispanic race/ethnicities compared to NH-white population were also reported in an earlier NHANES analysis [17]. The Second National Report on Biochemical Indicators of Diet and Nutrition in the U.S. Population [24] reported that in 2003–2006 about 10% of the U.S. population had nutrition deficiencies (based on nutrient-related biomarkers) which varied by age, gender, or race/ethnicity and could be as high as nearly one third of certain population groups. The lower intake of the “underconsumed nutrients” and “nutrients of public health concern” among NH-black and Hispanic population as observed in this and previous analyses may contribute, in part, to the disparities in diet-related diseases observed among these race/ethnic groups [39,40,41,42]. Efforts to better communicate dietary recommendations, possibly using customized cultural relevant messages, should be considered to help certain race/ethnic groups meet nutrient needs. The use of dietary supplements for some of these groups should also be considered.

A major strength of our study was the use of a large nationally representative population-based sample of adults. One of the limitations to our study was that the estimates relied on self-reported data for nutrient intakes and as such were subject to bias. The analysis, however, relied on the assumptions that 24-hour dietary recall-based nutrient intakes from food sources were unbiased and self-reported dietary supplement intake accurately reflected long-term intake patterns. Although the dietary supplement data were self-reported, NHANES interviewers verified the self-reporting accuracy by examining the dietary supplement bottles/labels 85% of the time [8]. Furthermore, estimates of vitamins and minerals contributed by dietary supplements relied on the label declarations rather than analytic values. The small sample size for NH-Asian population was another potential limitation. Finally, given the desire to compare across race/ethnicity groups, a large number of statistical comparisons were made which can increase possibility of Type II errors. The results of this study should be interpreted with these limitations in mind. 

In conclusion, the results of this study suggest an association between dietary supplement use and achieving adequate intakes of various nutrients in all race/ethnic groups, and that the association is stronger for NH-White population subgroup than other population subgroups. 

## Figures and Tables

**Table 1:** Usual intake of nutrients from foods and foods + dietary supplements among adults (19+ years old) by race/ethnicity. NHANES 2009–2012, gender combined data ^1^.

Nutrients	NH-White (*n* = 4649)		NH-Black (*n* = 2358)		Hispanic (*n* = 2625)		NH-Asian ** (*n* = 615)	
	Food Only	Food + Supplement	Food Only	Food + Supplement	Food Only	Food + Supplement	Food Only	Food + Supplement
Calcium (mg)	1057 ± 10	1244 ± 11 *	847 ± 13	935 ± 15 *	990 ± 17	1073 ± 19 *	788 ± 17	942 ± 22 *
Copper (mg)	1.35 ± 0.01	1.70 ± 0.02 *	1.20 ± 0.02	1.40 ± 0.02 *	1.27 ± 0.02	1.46 ± 0.03 *	1.44 ± 0.02	1.69 ± 0.05 *
Iron (mg)	15.8 ± 0.2	19.2 ± 0.2 *	14.1 ± 0.2	16.9 ± 0.3 *	15.8 ± 0.3	17.6 ± 0.3 *	14.4 ± 0.4	17.0 ± 0.5 *
Magnesium (mg)	319 ± 3	351 ± 4 *	271 ± 5	286 ± 5	312 ± 4	327 ± 5	323 ± 6	344 ± 7
Phosphorus (mg)	1455 ± 10	1466 ± 11	1264 ± 17	1267 ± 17	1459 ± 20	1463 ± 20	1278 ± 24	1280 ± 23
Selenium (µg)	113 ± 1	132 ± 1 *	111 ± 2	122 ± 2 *	119 ± 2	147 ± 18	122 ± 3	137 ± 4 *
Zinc (mg)	12.1 ± 0.1	16.8 ± 0.3 *	10.8 ± 0.3	13.2 ± 0.3 *	11.7 ± 0.2	13.8 ± 0.2 *	10.4 ± 0.2	13.8 ± 0.5 *
Vitamin A (µg RE)	697 ± 18	1078 ± 26 *	542 ± 20	745 ± 22 *	541 ± 11	726 ± 16 *	614 ± 24	902 ± 47 *
Thiamin (mg)	1.70 ± 0.10	6.41 ± 0.52 *	1.48 ± 0.02	3.04 ± 0.22 *	1.65 ± 0.02	3.44 ± 0.26 *	1.56 ± 0.04	4.80 ± 0.73 *
Riboflavin (mg)	2.31 ± 0.02	5.39 ± 0.39 *	1.74 ± 0.03	3.07 ± 0.17 *	2.02 ± 0.03	3.57 ± 0.22 *	1.80 ± 0.05	4.00 ± 0.55 *
Niacin (mg)	26.4 ± 0.2	38.1 ± 1.0 *	24.9 ± 0.3	29.8 ± 0.6 *	26.7 ± 0.4	31.0 ± 0.6 *	24.7 ± 0.8	33.1 ± 2.3 *
Folate DFE (µg)	574 ± 7	813 ± 8 *	475 ± 9	612 ± 11 *	564 ± 9	684 ± 12 *	561 ± 15	745 ± 25 *
Vitamin B_6_ (mg)	2.18 ± 0.02	6.04 ± 0.26 *	1.94 ± 0.03	3.89 ± 0.2 *	2.22 ± 0.04	4.15 ± 0.25 *	2.10 ± 0.06	4.50 ± 0.59 *
Vitamin B_12_ (µg)	5.60 ± 0.11	56.8 ± 4.0 *	4.76 ± 0.12	38.5 ± 6.0 *	4.92 ± 0.12	38.6 ± 6.8 *	4.62 ± 0.20	62.0 ± 9.7 *
Vitamin C (mg)	82.8 ± 2.3	177 ± 7 *	92.0 ± 2.3	139 ± 7 *	93.6 ± 2.9	132 ± 4 *	94.2 ± 4.5	178 ± 12 *
Vitamin D (µg)	5.11 ± 0.08	16.2 ± 0.9 *	3.97 ± 0.12	9.28 ± 0.36 *	4.68 ± 0.09	8.50 ± 0.34 *	4.71 ± 0.32	15.0 ± 1.0 *
Vitamin E (mg)	8.84 ± 0.11	29.7 ± 1.4 *	7.79 ± 0.19	17.4 ± 0.9 *	7.64 ± 0.19	17.5 ± 1.4 *	8.50 ± 0.28	19.6 ± 1.4 *
Vitamin K (µg)	112 ± 3	119 ± 3	99.4 ± 3.6	104 ± 4	82.1 ± 3.1	87.0 ± 2.7	149 ± 10	157 ± 10
Choline (mg)	340 ± 3	341 ± 3	326 ± 4.2	326 ± 4	348 ± 6	350 ± 6	329 ± 8	330 ± 7

^1^ Mean ± standard error; * significantly different from Food Only column at *p* < 0.01; ** NHANES 2011–2012 only.

**Table 2:** Percent of adult (19+ years old) population below Estimated Average Requirement (EAR) or above Adequate Intake (AI) of nutrients from foods and foods + dietary supplements by race/ethnicity. ^1^ NHANES 2009–2012 gender combined data.

Nutrients	NH-White (*n* = 4649)		NH-Black (*n* = 2358)		Hispanic (*n* = 2625)		NH-Asian ** (*n* = 615)	
	Food Only	Food + Supplement	Food Only	Food + Supplement	Food Only	Food + Supplement	Food Only	Food + Supplement
Nutrients with EAR, percentage below EAR								
Calcium	35.9 ± 1.0 ^1^	24.2 ± 0.7 *	56.3 ± 1.4	48.2 ± 1.4 *	36.4 ± 2.1	30.1 ± 2.0	61.4 ± 2.5	47.0 ± 2.6 *
Copper	4.5 ± 0.6	3.6 ± 0.4	6.7 ± 1.3	5.7 ± 1.1	5.6 ± 1.4	4.9 ± 1.3	2.4 ± 0.9	2.0 ± 0.7
Iron	3.1 ± 0.2	2.3 ± 0.2	5.5 ± 0.5	4.6 ± 0.4	5.2 ± 0.5	4.5 ± 0.5	7.0 ± 1.3	5.6 ± 1.1
Magnesium	48.5 ± 1.3	40.3 ± 1.2 *	67.2 ± 2.2	61.7 ± 1.9	51.3 ± 2.0	46.7 ± 2.1	46.1 ± 2.4	40.1 ± 2.5
Phosphorus	<1	<1	<1	<1	<1	<1	1.2 ± 0.5	1.3 ± 0.5
Selenium	<1	<1	<1	<1	<1	<1	<1	<1
Zinc	11.9 ± 1.3	8.2 ± 1.0	22.1 ± 2.5	17.9 ± 2.0	15.4 ± 2.0	13.0 ± 2.0	20.6 ± 1.8	15.8 ± 1.7
Vitamin A	37.7 ± 2.0	27.2 ± 1.3 *	59.7 ± 3.0	49.0 ± 2.5 *	59.0 ± 2.0	51.0 ± 1.8 *	45.3 ± 5.6	33.9 ± 4.0
Thiamin	4.1 ± 0.6	2.6 ± 0.4	10.1 ± 1.2	8.1 ± 1.0	7.7 ± 1.5	6.3 ± 1.3	5.8 ± 1.4	4.1 ± 1.2
Riboflavin	1.3 ± 0.2	<1	6.8 ± 1.3	5.6 ± 1.1	4.4 ± 1.1	3.9 ± 0.9	9.0 ± 1.4	6.8 ± 1.3
Niacin	<1	<1	1.4 ± 0.5	1.2 ± 0.4	1.6 ± 0.5	1.3 ± 0.5	1.1 ± 0.7	<1
Folate DFE	9.1 ± 1.0	5.9 ± 0.6 *	19.0 ± 1.6	14.9 ± 1.3	9.0 ± 1.4	7.8 ± 1.3	8.5 ± 1.6	6.1 ± 1.3
Vitamin B_6_	9.2 ± 0.7	5.9 ± 0.5 *	11.2 ± 1.7	8.8 ± 1.3	7.7 ± 1.7	6.2 ± 1.4	7.90 ± 2.23	5.4 ± 1.6
Vitamin B_12_	3.2 ± 0.6	1.9 ± 1.4	3.4 ± 1.5	2.5 ± 1.1	4.9 ± 0.9	3.9 ± 0.8	3.9 ± 2.8	2.6 ± 1.7
Vitamin C	46.9 ± 1.6	32.6 ± 1.4 *	37.4 ± 2.8	30.7 ± 2.2	38.4 ± 2.5	32.3 ± 2.2	30.1 ± 6.2	23.5 ± 4.2
Vitamin D	93.2 ± 0.6	57.9 ± 0.8 *	98.5 ± 0.7	77.2 ± 1.0 *	96.9 ± 0.6	80.1 ± 0.9 *	95.1 ± 1.7	65.7 ± 2.4 *
Vitamin E	82.6 ± 0.9	54.6 ± 0.8 *	90.3 ± 1.8	71.5 ± 1.8 *	91.2 ± 1.7	75.3 ± 1.8 *	85.8 ± 3.3	62.6 ± 3.8 *
Nutrients with AI, percentage above AI								
Vitamin K	44.6 ± 1.5	50.0 ± 1.5	38.2 ± 2.5	41.5 ± 2.3	24.0 ± 2.6	27.8 ± 2.3	69.0 ± 5.7	71.9 ± 5.1
Choline	8.2 ± 0.8	8.5 ± 0.8	5.9 ± 1.1	6.0 ± 1.1	9.5 ± 1.5	9.9 ± 1.8	4.2 ± 1.4	4.2 ± 1.5

^1^ Mean ± standard error; * significantly different from Food Only column at *p* < 0.01; ** NHANES 2011–2012 only.

**Table 3:** Comparison of percent adult (19+ years old) population below Estimated Average Requirement (EAR) or above Adequate Intake (AI) of nutrients from foods + dietary supplements among different race/ethnicity groups. ^1^ NHANES 2009–2012 gender combined data.

Nutrients	NH-White (*n* = 4649)	NH-Black (*n* = 2358)	Hispanic (*n* = 2625)	NH-Asian * (*n* = 615)
Nutrients with EAR, percentage below EAR				
Calcium	24.2 ± 0.7 ^1^ a	48.2 ± 1.4 b	30.1 ± 2.0 c	47.0 ± 2.6 b
Copper	3.6 ± 0.4 ab	5.73 ± 1.12 a	4.92 ± 1.28 ab	1.95 ± 0.71 b
Iron	2.3 ± 0.18 a	4.59 ± 0.44 b	4.47 ± 0.47 b	5.63 ± 1.11 b
Magnesium	40.3 ± 1.2 a	61.7 ± 1.9 b	46.7 ± 2.1 c	40.1 ± 2.5 ac
Phosphorus	<1	<1	<1	1.3 ± 0.5
Selenium	<1	<1	<1	<1
Zinc	8.2 ± 1.0 a	17.9 ± 2.0 b	13.0 ± 2.0 ab	15.8 ± 1.7 b
Vitamin A	27.2 ± 1.3 a	49.0 ± 2.5 b	51.0 ± 1.8 c	33.9 ± 4.0 a
Thiamin	2.6 ± 0.4 a	8.1 ± 1.0 b	6.3 ± 1.3b c	4.1 ± 1.2 ac
Riboflavin	<1a	5.61 ± 1.13 b	3.85 ± 0.85 b	6.79 ± 1.28 b
Niacin	<1	1.2 ± 0.4	1.3 ± 0.5	<1
Folate DFE	5.9 ± 0.6 a	14.9 ± 1.3 b	7.8 ± 1.3 a	6.1 ± 1.3 a
Vitamin B_6_	5.9 ± 0.5	8.8 ± 1.3	6.2 ± 1.4	5.4 ± 1.6
Vitamin B_12_	1.9 ± 1.4	2.5 ± 1.1	3.9 ± 0.8	2.6 ± 1.7
Vitamin C	32.6 ± 1.4	30.7 ± 2.2	32.3 ± 2.2	23.5 ± 4.2
Vitamin D	57.9 ± 0.8 a	77.2 ± 1.0 b	80.1 ± 0.9 b	65.7 ± 2.4 c
Vitamin E	54.6 ± 0.8 a	71.5 ± 1.8 b	75.3 ± 1.8 c	62.6 ± 3.8 ab
Nutrients with AI, percentage above AI				
Vitamin K	50.0 ± 1.5 a	41.5 ± 2.3 b	27.8 ± 2.3 c	71.9 ± 5.1 d
Choline	8.5 ± 0.8	6.0 ± 1.1	9.9 ± 1.8	4.9 ± 1.5

^1^ Mean ± standard error; values with different letters in a row indicates significant differences across race/ethnicity groups at *p* < 0.01. * NHANES 2011–2012 only.

**Table 4:** Percent adult (19+ years old) population exceeding Tolerable Upper Limit of intake (UL) of nutrients from foods and foods + dietary supplements by race/ethnicity. ^1^ NHANES 2009–2012 gender combined data.

Nutrients	NH-White (*n* = 4649)		NH-Black (*n* = 2358)		Hispanic (*n* = 2625)		NH-Asian ** (*n* = 615)	
	Food Only	Food + Supplement	Food Only	Food + Supplement	Food Only	Food + Supplement	Food Only	Food + Supplement
Calcium	<1	4.8 ± 0.3 *^,1^	<1	1.0 ± 0.3	<1	1.1 ± 0.2 *	<1	<1
Copper	<1	<1	<1	<1	<1	<1	<1	<1
Iron	<1	1.9 ± 0.2 *	<1	1.9 ± 0.3 *	<1	1.1 ± 0.2 *	<1	1.2 ± 0.5
Phosphorus	<1	<1	<1	<1	<1	<1	<1	<1
Selenium	<1	<1	<1	<1	<1	<1	<1	<1
Zinc	<1	2.5 ± 0.3 *	<1	<1	<1	<1	<1	1.5 ± 0.6
Vitamin A	<1	<1	<1	<1	<1	<1	<1	<1
Niacin	ND	ND	ND	ND	ND	ND	ND	ND
Folate DFE	<1	2.1 ± 0.2 *	<1	<1	<1	1.3 ± 0.3 *	<1	<1
Vitamin B_6_	<1	1.1 ± 0.2 *	<1	<1	<1	<1	<1	<1
Vitamin C	<1	<1	<1	<1	<1	<1	<1	<1
Vitamin D	<1	1.5 ± 0.2 *	<1	<1	<1	<1	<1	2.2 ± 0.7 *
Vitamin E	<1	<1	<1	<1	<1	<1	<1	<1
Choline	<1	<1	<1	<1	<1	<1	<1	<1

^1^ Mean ± standard error; * significantly different from Food Only at *p* < 0.01; ** NHANES 2011–2012 only. Vitamin A, folate and vitamin E ULs based on retinol, folic acid, and added alpha tocopherol, respectively. ND: Not determined as niacin UL is based on a particular form of niacin (nicotinic acid) which is not quantified in NHANES.
